# Finding the optimal candidate for shock wave lithotripsy: external validation and comparison of five prediction models

**DOI:** 10.1007/s00240-023-01444-4

**Published:** 2023-04-07

**Authors:** Marcin Popiolek, Johan Jendeberg, Pernilla Sundqvist, Magnus Wagenius, Mats Lidén

**Affiliations:** 1 Department of Urology, Faculty of Medicine and Health, Örebro University, 701 85 Örebro, Sweden; 2 Department of Radiology, Faculty of Medicine and Health, Örebro University, Örebro, Sweden; 3 Department of Clinical Sciences, Division of Infection Medicine, Lund University, Lund, Sweden; 4 Department of Urology Helsingborg Hospital, Helsingborg, Sweden

**Keywords:** Shock wave lithotripsy, Ureteral stones, Nomograms, Validation, Outcomes

## Abstract

We aimed to externally validate five previously published predictive models (Ng score, Triple D score, S_3_HoCKwave score, Kim nomogram, Niwa nomogram) for shock wave lithotripsy (SWL) single-session outcomes in patients with a solitary stone in the upper ureter. The validation cohort included patients treated with SWL from September 2011 to December 2019 at our institution. Patient-related variables were retrospectively collected from the hospital records. Stone-related data including all measurements were retrieved from computed tomography prior to SWL. We estimated discrimination using area under the curve (AUC), calibration, and clinical net benefit based on decision curve analysis (DCA). A total of 384 patients with proximal ureter stones treated with SWL were included in the analysis. Median age was 55.5 years, and 282 (73%) of the sample were men. Median stone length was 8.0 mm. All models significantly predicted the SWL outcomes after one session. S_3_HoCKwave score, Niwa, and Kim nomograms had the highest accuracy in predicting outcomes, with AUC 0.716, 0.714 and 0.701, respectively. These three models outperformed both the Ng (AUC: 0.670) and Triple D (AUC: 0.667) scoring systems, approaching statistical significance (*P* = 0.05). Of all the models, the Niwa nomogram showed the strongest calibration and highest net benefit in DCA. To conclude, the models showed small differences in predictive power. The Niwa nomogram, however, demonstrated acceptable discrimination, the most accurate calibration, and the highest net benefit whilst having relatively simple design. Therefore, it could be useful for counselling patients with a solitary stone in the upper ureter.

## Introduction

With an incidence of 5–13% worldwide, urolithiasis is a rising global concern and a significant burden for healthcare systems [1, 2]. Epidemiological data have shown upward trends in both the prevalence of and interventions for kidney stone disease [3, 4]. Most ureteral stones (75–90%) pass spontaneously with no need for intervention and low morbidity [5]. Thus, majority of ureteral stones can be managed conservatively if there are no indications for active removal such as persistent pain, obstruction, or kidney failure [6]. However, stones in the upper ureter have a lower likelihood of spontaneous passage (22–48%) than those in other locations [7, 8] and their management may be especially challenging if they become impacted.

The European Association of Urology (EAU) considers both shock wave lithotripsy (SWL) and ureteroscopy (URS) first-line treatment options yielding similar outcomes for ureteral stones under 10 mm [6].

Although SWL is less invasive than URS and unlike other treatment modalities does not require general anaesthesia, a wide range of factors described in the literature may influence its efficacy [9]. Factors affecting SWL outcomes include stone size measured as one-dimensional stone length or stone volume, location, density, and skin-to-stone distance (SSD) [10–14]. Furthermore, parameters measured on computed tomography (CT) scans indicating an impacted ureteral stone also seem to be valuable predictors of SWL failure [15, 16].

Proximal ureteral stones often pose a challenge for the clinician to choose the most suitable treatment option. Since patient selection is key to successful SWL, numerous attempts have been made to develop a reliable predicting scoring model or nomogram to enhance clinical decision-making [17–21]. Although several predictive models are available, a solid ground for decision-making is still missing. A comparative external validation of the models may provide that missing ground.

The aim of this study was therefore to externally validate and compare the predictive scoring models and nomograms available in the literature and to evaluate their performances in terms of discrimination, calibration, and clinical usefulness in predicting SWL outcomes after one session.

## Material and methods

### Patient population (validation cohort)

**Fig. 1:**
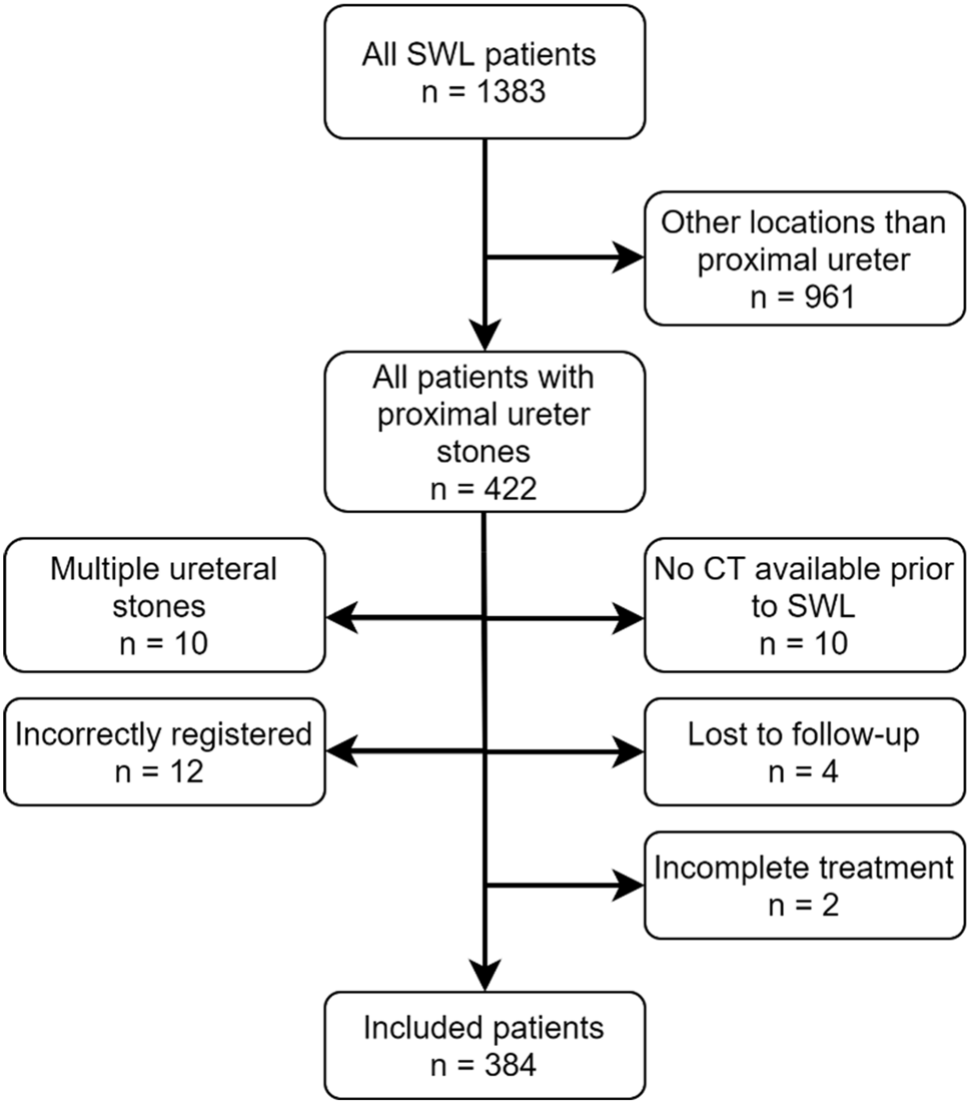
Flowchart showing exclusion criteria and numbers

Ethical approval was obtained from the Swedish Ethical Review Authority (2019-04689). We conducted a retrospective review of all 1383 consecutive patients treated with SWL in Örebro University Hospital in Sweden from September 2011 to December 2019. Exclusion criteria and numbers are shown in the flowchart (Fig. 1). Of the total sample, 384 were eligible for inclusion in the study. Patient characteristics are presented in Table 1.

**Table 1:** Patient characteristics and stone parameters measured on computer tomography (CT)

	All patients (n = 384)	Successful SWL (n = 228)	Failed SWL (n = 156)	*P* value
Age (yrs): median (IQR)	55.5 (45.0–67.7)	54.5 (41.5–65.0)	64.0 (50.0–70.0)	< 0.001
Sex: n (%)				
Male	282 (73%)	155 (68%)	127 (81%)	0.003
Female	102 (27%)	73 (32%)	29 (19%)	
Stone length (mm): median (IQR)	8.0 (6.4–9.9)	7.3 (6.2–8.9)	9.3 (7.6–11.2)	< 0.001
Ellipsoid stone volume (mm^3^): median (IQR)	138 (82–225)	112 (74–169)	198 (117–338)	< 0.001
Average CT attenuation (HU): mean ± SD	952 ± 255	902 ± 16	1025 ± 20	< 0.001
Maximal CT attenuation (HU), mean ± SD	1213 ± 288	1167 ± 18	1281 ± 23	< 0.001
Skin-to-stone distance (cm): mean ± SD				
At 90º	13.0 ± 1.9	12.8 ± 1.9	13.3 ± 2.0	0.007
At 45º	13.9 ± 2.0	13.6 ± 2.0	14.2 ± 2.1	0.006
Hydronephrosis, n (%)				
No	46 (12%)	35 (15%)	11 (7%)	< 0.001
Grade 1	223 (58%)	146 (64%)	77 (49%)	
Grade 2	91 (24%)	42 (18%)	49 (31%)	
Grade 3	19 (5%)	5 (2%)	14 (9%)	
Grade 4	5 (1%)	0 (0)	5 (3%)	
Stone-free, n (%)	228 (59%)^a^			

*IQR* interquartile range *SD* standard deviation, *HU* Hounsfield units, *CT* computed tomography

^a^Number of stone free patients after one SWL session

### Patient data

Clinical data such as age, sex, number of SWL sessions, and treatment outcomes were collected from patients’ medical records. Stone-related data were obtained from CT scans using the integrated PACS measurement tool (Sectra IDS7, Linköping, Sweden).

### CT scans and measurement methodology

All patients were examined with CT before SWL. Typically, a low-dose unenhanced protocol for urinary tract stones was followed using similar settings (120 kV, CTDI 2–5 mGy). Axial, coronal, and sagittal reformations (3 or 5 mm) were generated. The same experienced urologist (MP) performed all measurements under supervision and in consensus with expert radiologists (ML, JJ). The reader was not aware of SWL outcomes at the time of measurements.

All measurements were performed in a standardized soft-tissue window (C50, W400) with selected zoom level (pixel-to-pixel × 6–8). A special care was devoted to measurements of attenuation in small stones (stone length < 5 mm) as obtaining precise values is not always straightforward in these cases. To increase accuracy, we took several measurements on different CT slices in high zoom level and, if necessary, a bone window (C400, W1500) was used to assess stone structure and identify areas with highest attenuation. The largest diameter of the stone was obtained in all reformations (axial, coronal, and sagittal) and the longest was defined as the stone length. The measurements were reported in mm to one decimal place. The stone volume was estimated with the ellipsoid formula using stone diameters in three axes measured on coronal and axial reformations [22]. SSD was measured on axial reformation dorsally from the centre of the stone to the skin in both vertical (SSD 90°) and oblique (SSD 45°) directions. The presence of hydronephrosis was graded 0–4 (0 = none, 1 = mild, 2 = moderate, 3 = pronounced, 4 = massive) [23]. Proximal ureter was defined as the segment between the ureteropelvic junction and the level of ureter projecting over the upper border of the sacroiliac joint.

### Shock wave lithotripsy

SWL was performed using Siemens Lithostar Modularis (Siemens AG, Erlangen, Germany) under fluoroscopic control. The patient received suppository diclofenac 100 mg prior to treatment. During the procedure, a combination of intravenous alfentanil and propolipid was administered intermittently in small doses to provide analgesia and sedation. According to the local protocol, stones in the ureter within the level of the kidney parenchyma were treated with a maximum energy of 4 kV and 4000 shockwaves. Stones below the parenchyma were treated with maximal energy of 6 kV and 2500 shockwaves. A frequency of 60 shock waves per minute (1 Hz) was used in all cases. All patients were treated in supine position with the shock wave head placed obliquely underneath the table. Treatment outcome was evaluated with follow-up imaging (kidney, ureter, and bladder radiograph [n = 22], antegrade pyelogram [n = 10], intravenous urography [n = 68], and CT [n = 284]) at 2 to 6 weeks depending on the presumed results of the first session. Stone-free status was defined as no stones in the ureter (zero residuals) on follow-up examination.

### Predictive models

We performed a literature search for nomograms and scoring systems predicting SWL outcomes based on information from CT after one session in patients with proximal ureteral stones. Five predictive models that fulfilled these criteria (Ng score, Triple D score, S_3_HoCKwave score, Niwa nomogram, Kim nomogram) were included in the study for external validation [17–21]. The variables included in each model are summarized in Table 2.

**Table 2:** Variables included by the different scoring systems and nomograms

	Ng score	Triple D score	S3HoCKwave score	Kim nomogram	Niwa nomogram
Sex			X	X	
Number of stones				X	
Stone location			X	X	
Stone length			X	X	X
Stone volume	X	X			
Stone density	X^a^	X^a^	X^a^	X^a^	X^b^
SSD	X^c^	X^d^	X^e^		X^c^
Colic			X		
Hydronephrosis grade (0–4)				X	

^a^Average CT attenuation (HU)

^b^Maximum CT attenuation (HU)

^c^SSD 90°

^d^SSD mean of three measurements (0°, 45°, 90°)

^e^Unknown methodology

### Sample size calculation

Steyerberg et al. recommended as a rule of thumb at least 100 events and 100 non-events in an external sample to achieve a reasonable power for statistical analysis to validate a prediction model [24]. According to the results of an internal audit at our institution, the success rates in proximal ureteral stones after one SWL session ranged from 50 to 70%. Based on these numbers, we estimated that the validation cohort should include 200 to 350 study subjects.

### Statistics

The statistical analysis was performed using IBM SPSS v27.0.1.0 (SPSS Inc., Chicago, IL, USA) and Stata MP 17.0 (StataCorp, Texas, USA). The parameters in our study were analysed using absolute and relative frequencies for quantitative variables. Continuous variables are reported as mean and standard deviation (SD) or median and interquartile range (IQR). Pearson’s chi-square or Fisher exact test was used to verify associations between quantitative variables. Between-group comparisons for qualitative variables were performed using student *T* test or Mann–Whitney *U* test. To calculate the predicted probability of successful SWL, we used the beta-coefficients from regression models provided by the authors (Niwa nomogram, Kim nomogram, and S_3_HoCKwave scores). We used published estimates to validate Triple D and Ng scores and performed the validation process following TRIPOD’s recommended steps [25].

To assess the discrimination capability of the models, we calculated and compared the areas under the curves (AUC) of the receiver operator characteristics (ROC). Calibration was assessed by visual representation of the relationship between the predicted and observed values using a flexible curve on calibration plots. Additional calibration measures such as intercept and slope were also included. Calibration intercept is an assessment of calibration-in-the-large (CITL) and has a target value of zero [26]. Values below zero suggest overestimation; those over zero suggest underestimation. Calibration slope is a measure of the spread of an estimate and has a target value of 1. When CITL is close to zero, a slope close to 1 indicates good calibration across all subgroups. Decision curve analysis (DCA) was performed to estimate the net benefit of the models with regard to different clinically relevant thresholds [27]. A two-sided *P* < 0.05 was considered statistically significant.

## Results

### Validation cohort

Baseline patient and stone characteristics for the validation cohort are summarized in Table 2. Median age was 55.5 years (IQR: 45–68) and 282 (73%) were males. Median stone length was 8.0 mm (IQR: 6.4–9.9), and median estimated volume was 138 mm^3^ (IQR: 82–225). Mean average and maximal CT attenuation were 952 ± 255 HU and 1213 ± 288 HU, respectively. Mean SSD was 13.0 ± 1.9 cm at 90 degrees and 13.9 ± 2.0 cm at 45 degrees. Stone-free status was achieved in 228 (59%) patients after one session and in 298 (78%) after all sessions (range 1–4, mean 1.36). Overall, approximately 30% had hydronephrosis grade 2–4. A comparison of the successfully treated group and patients with failed SWL outcome is presented in Table 1.

### Discrimination

**Fig. 2:**
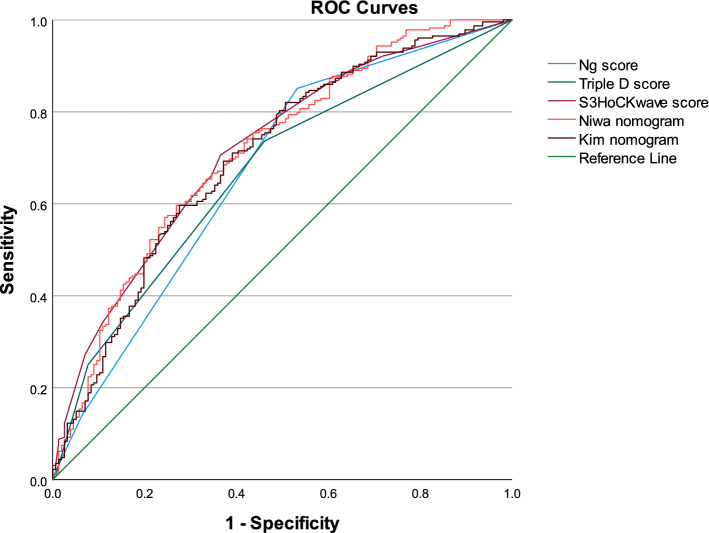
ROC curves

The discriminatory capacity of the model is visually presented on ROC plots (Fig. 2). All the models significantly predicted the SWL outcomes after one session. S_3_HoCKwave score, Niwa and Kim nomogram had highest accuracy for prediction of successful outcome with AUC 0.716 (95% CI 0.664–0.769), 0.714 (95% CI 0.661–0.766) and 0.701 (95% CI 0.648–0.755), respectively. These three models outperformed Ng (AUC: 0.670; 95% CI 0.614–0.726) and Triple D (AUC: 0.667; 95% CI 0.612–0.721) scoring systems’ discrimination power. Sensitivity, specificity and accuracy for probability threshold of 50% are presented in Table 3.The pairwise differences between the Ng score or Triple D score and S3oCKwave score, Niwa nomogram, Kim nomogram were statistically significant (*P* < 0.05). However, there was no significant difference between and S3oCKwave score, Niwa nomogram and Kim nomogram (*P* = 0.64).

**Table 3:** Measures of performance for different prediction models

Prediction model(s)	Sensitivity^a^	Specificity^a^	Accuracy^a^	AUC	95% CI
S_3_HoCKwave score	161/228 (71%)	99/156 (63%)	260/384 (68%)	0.716	0.664–0.768
Niwa nomogram	174/228 (76%)	84/156 (54%)	258/384 (67%)	0.714	0.661–0.766
Kim nomogram	174/228 (76%)	82/156 (53%)	256/384 (66%)	0.701	0.648–0.755
Ng score	32/228 (14%)	146/156 (93%)	178/384 (46%)	0.670	0.614–0.726
Triple D score	57/228 (28%)	155/156 (99%)	212/384 (55%)	0.667	0.612–0.721

*AUC* area under the curve, *CI* confidence interval for AUC

^a^Calculated using 50% probability threshold

### Calibration

**Fig. 3:**
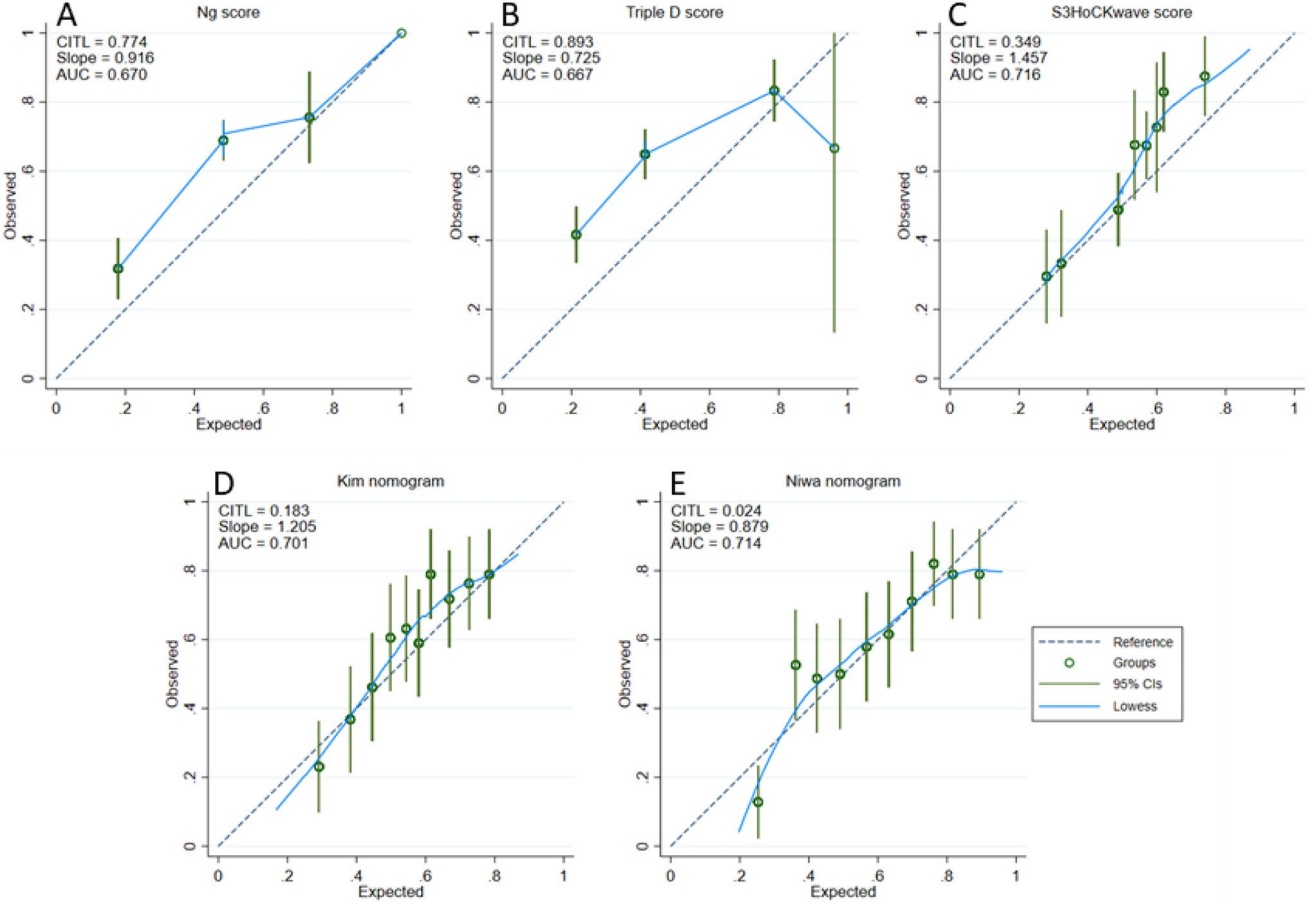
Calibration plots: **A** Ng score **B** Triple D score **C** S_3_HoCKwave score **D** Kim nomogram** E** Niwa nomogram

Calibration plots including key measures are presented in Fig. 3. Of all models, Ng score and Triple D score showed the weakest overall calibration with CITLs of 0.774 and 0.893, respectively. Both S_3_HoCKwave score and Kim nomogram had CITLs above zero (0.349 and 0.183), indicating slight systematic underestimation of these models’ predictions. The Niwa nomogram showed the strongest calibration of all models, with a CITL close to zero (0.024). However, the calibration slope for this model was 0.879, suggesting it had weaker calibration in some subgroups.

### Decision curve analysis (DCA): net benefit

**Fig. 4:**
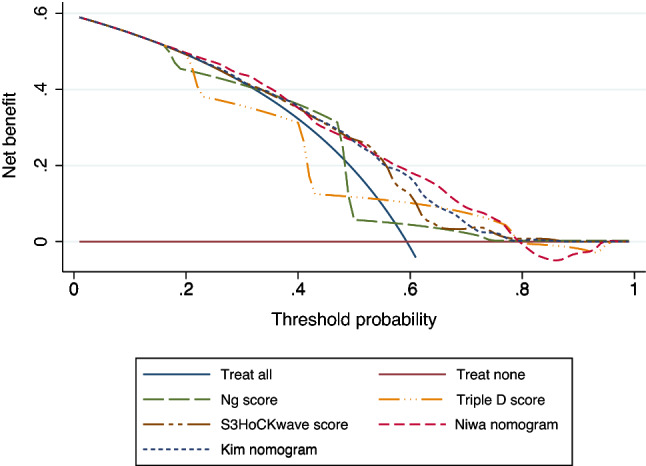
Decision curve analysis: net benefit

In the DCA, Ng scores and Triple D scores showed no net benefit as their decision curves oscillated close to the “treat all strategy” curve (Fig. 4). In contrast, S_3_HoCKwave score and both Kim and Niwa nomograms provided a net benefit, over treating all patients or none of them. Of these three, the Niwa nomogram showed the highest net benefit over the widest range of thresholds (20–80%).

## Discussion

In this study, we compared the performance of five prediction models for SWL outcomes after one session in an independent retrospective cohort. The models showed moderate predictive capacity. There were small, but statistically significant, differences in discriminative AUC, calibration, and net benefit according to DCA. In our cohort, Ng score and Triple D score showed slightly lower discrimination (AUC 0.660 and 0.667, respectively) and poorer calibration, making them less useful in clinical praxis.

Yoshioka et al. tested the Triple D score performance and found that the AUC for this model in their cohort was 0.68, which was strikingly similar to AUC for this model in our study (0.67) but differed greatly from that reported in the original paper (0.78). Although S_3_HoCKwave score, Kim nomogram, and Niwa nomogram performed slightly better in terms of discrimination, the AUCs for these models were only moderate. There was no significant difference between these three latter models (*P* = 0.64). Kim nomogram and S_3_HoCKwave score also demonstrated some miscalibration, which diminished their net benefit. Niwa nomogram, however, showed adequate calibration and the highest net benefit. In addition, it includes fewer parameters than S_3_HoCKwave score or Kim nomogram. Furthermore, maximum stone attenuation and vertical SSD used in this model are easier to obtain than mean stone attenuation and oblique SSD and are less dependent on the reader.

Recent improvements in ureteroscopy such as new laser technologies and new ureteroscopes with high quality vision have led over the last decade to a rapid increase in its popularity over that of SWL treatment [28]. Yet, SWL is still a viable treatment option for upper urinary tract stones [29]. Proximal ureteral stones may be challenging to manage if they become impacted. According to EAU Urolithiasis Guidelines, stones with a low likelihood of spontaneous passage should be considered for early intervention. However, the panel concludes that no exact cut-off values for stone size can be provided due to a lack of evidence. EAU guidelines recommend either SWL or URS to manage proximal ureteral stones sized < 10 mm, but favour neither over the other [6] and which is preferable remains controversial. A recently published RCT found that SWL for ureteric stones is the more cost-effective option but requires more treatments than URS and results in a lower quality of life [30]. According to Cone et al., a stone-free rate of at least 60% after one session should be achieved with SWL to consider it a cost-effective treatment compared with URS. This implies the necessity of adequate patient selection for SWL to be the most cost-effective overall.

Non-contrast CT is frequently used prior to SWL to assess the patient and stone-related factors influencing stone-free rate [31]. Several reports show that factors such as stone size, stone CT attenuation, and SSD are strong predictors of various SWL outcomes. Longest diameter of the stone is a proxy parameter of stone burden. Choi et al. found that stone size was an independent predictive factor influencing the outcomes of SWL [14]. In a study by Wagenius et al., stone size and age were found to be independent predictive factors for SWL failure [12]. Ng et al. observed that SSD measured vertically in patients with stones in the upper ureter had a significant predictive value for SWL outcomes [18]. Stone CT attenuation is one of the most cited predictive factors. Ouzaid et al. found that SFR dropped from 96 to 38% for stones with mean stone attenuation of ≥ 970 HU [13]. In contrast, Niwa et al. demonstrated that maximum CT attenuation value was a more significant predictor than mean attenuation [19]. None of these factors alone, however, is powerful enough to base a clinical decision on, as the SWL result is the product of a complex process with multifactorial interactions. Thus, predictive tools, such as nomograms and scoring systems, combining different predictors, have been developed to facilitate clinical decision-making and avoid unnecessary procedures.

To our knowledge, this is the first study to externally validate and compare several predictive models for SWL outcomes in proximal ureteral stones. According to our findings, the Niwa nomogram seems to be the most beneficial of all validated models and therefore may be considered an interesting candidate for use in decision-making with patients with solitary calculi in the upper ureter. However, given that the discrimination accuracy was only slightly above 0.7 (AUC), there is still room for improvement. Recently, Bulbul et al. showed that increased ureteral wall thickness on the CT scans was an independent predictive factor for SWL failure [15]. It is likely that including ureteral wall thickness into predictive models, as a measure of potential stone impaction, may increase the models’ discrimination power.

Our study has some limitations. Its retrospective methodology increases the risk of misclassification and selection bias and reflects only those patients already selected for SWL at the study site. Moreover, not all consecutive patients managed at our institution with proximal ureteral stones, suitable for active treatment, underwent SWL because stones > 10 mm were preferably treated with URS in line with EAU Urolithiasis Guidelines. The patients underwent CT on several different scanners with slightly different protocols, which may have had an impact on the measurements and presents another risk of possibly skewed results. However, this variation in scanners may also reflect real-world better than strictly controlled data. A multicentre study would also increase the generalizability of the results of this single-centre study. Nonetheless, all the treatments were executed with methodological consistency according to the local protocols for shock wave rate, energy, and sedation/analgesia regime, which improves the validity of this study. Lastly, although all measurements were performed by only one reader, which increases the risk of systematic error, they were done under supervision and according to the methodology described in detail in the study protocol.

## Conclusions

In our cohort, although there were small differences, the Niwa nomogram showed acceptable discrimination compared with the other methods, the most accurate calibration, and the highest net benefit with a relatively simple design. It could therefore be useful in counselling patients with a solitary stone in the upper ureter during the decision-making process. Recalibration of other models could be considered in future studies to increase their accuracy and clinical usefulness.

## Data Availability

The datasets generated and/or analysed during the current study are not publicly available due to current data protection legislation, but are available from the corresponding author on reasonable request, if appropriate permits are obtained from adequate authorities.
